# A Novel High Temperature Fuel Cell Proton Exchange Membrane with Nanoscale Phase Separation Structure Based on Crosslinked Polybenzimidazole with Poly(vinylbenzyl chloride)

**DOI:** 10.3390/nano13020266

**Published:** 2023-01-07

**Authors:** Erli Qu, Min Xiao, Dongmei Han, Sheng Huang, Zhiheng Huang, Wei Liu, Shuanjin Wang, Yuezhong Meng

**Affiliations:** 1 The Key Laboratory of Low-Carbon Chemistry & Energy Conservation of Guangdong Province/State Key Laboratory of Optoelectronic Materials and Technologies, School of Materials Science and Engineering, Sun Yat-sen University, Guangzhou 510275, China; 2 School of Chemical Engineering and Technology, Sun Yat-Sen University, Zhuhai 519000, China; 3 Research Center of Green Catalysts, College of Chemistry, Zhengzhou University, Zhengzhou 450001, China; 4 Institute of Chemistry, Henan Academy of Sciences, Zhengzhou 450000, China

**Keywords:** polybenzimidazole, crosslinked membrane, high temperature proton exchange membrane, high temperature fuel cell

## Abstract

A semi-aromatic polybenzimidazole (DPBI) is synthesized via polycondensation of decanedioic acid (DCDA) and 3,3-diaminobenzidine (DAB) in a mixed phosphorus pentoxide/methanesulfonic acid (PPMA) solvent. Ascribing to in-situ macromolecular crosslinker of ploly((vinylbenzyl chloride) (PVBC), a robust crosslinked DPBI membrane (DPBI-*x*PVBC, *x* refers to the weight percentage of PVBC in the membrane) can be obtained. Comprehensive properties of the DPBI and DPBI-*x*PVBC membranes are investigated, including chemical structure, antioxidant stability, mechanical strength, PA uptake and electrochemical performances. Compared with pristine DPBI membrane, the PA doped DPBI-*x*PVBC membranes exhibit excellent antioxidative stability, high proton conductivity and enhanced mechanical strength. The PA doped DPBI-*10*PVBC membrane shows a proton conductivity of 49 mS cm^−1^ at 160 °C without humidification. Particularly, it reveals an enhanced H_2_/O_2_ single cell performance with the maximum peak power density of 405 mW cm^−2^, which is 29% higher than that of pristine DPBI membrane (314 mW cm^−2^). In addition, the cell is very stable in 50 h, indicating the in-situ crosslinked DPBI with a macromolecular crosslinker of PVBC is an efficient way to improve the overall performance of HT-PEMs for high performance HT-PEMFCs.

## 1. Introduction

High temperature proton exchange membrane fuel cells (HT-PEMFCs, >120 °C) have been recently attracting extensive attention of researchers due to their significant advantages, such as simplified water-heat systems, high CO tolerance, enhanced electrode reaction kinetics, and so on [1,2,3,4,5,6]. Proton exchange membrane (PEM) is one of the key components of HT-PEMFCs. The common available poly[(2,2′-(m-phenylene)-5,5′-(bibenzimidazole)] (*m*-PBI) has become a research hotspot due to its excellent thermal-chemical stability, mechanical properties, high glass transition temperature (T*_g_*), and excellent proton conduction performance after doping phosphoric acid (PA), but it has some shortcomings limiting its application such as high cost, poor solubility and hard processing formability [7,8,9,10,11,12,13]. On the other hand, the synthesis of PBI by conventional solution polymerization in polyphosphoric acid (PPA) is complicated with a multiple-step batch reaction procedure, which is unpractical to large-scale industrial production [14,15,16]. Furthermore, the plasticizing effect of PA on PBI leads to poor mechanical properties with high PA adsorption capacity, and the serious PA loss during fuel cell operation is also needed to be solved urgently [17,18,19,20]. In order to overcome these drawbacks, a number of approaches have been brought forward. Yang et al. [21] have reported several alternative synthetic methods to prepare *m*-PBI, among them, the microwave irradiation is an effective method to greatly shorten the polycondensation time in PPA. For example, Yang et al. [22] prepared a copolymer of polybenzimidazoles in PPA at 200 °C, and the process could be carried out within 90~110 min by means of microwave irradiation. However, the preparation of PBI through PPA process needs higher temperature (>170 °C) [23,24]. Recently, another reagent composed of phosphorus pentoxide-methane sulfonic acid (P_2_O_5_-MeSO_3_H) were proved to be a convenient alternative to overcome the difficulties from PPA [25,26,27,28]. Due to the highly viscous of PPA, it is difficult to stir effectively or operate conveniently even at high temperature, resulting in difficult scale up. Moreover, some organics are only slightly soluble in PPA, and even in any case, the dissolution rate is very low. 

At present, PEMs applied in HT-PEMFCs are all highly rigid aromatic series [29], especially commercial m-PBI, which has poor solubility and is difficult to process. In order to address these problems of PBI, appropriate structural modifications can be carried out on the polymer backbone. The introduction of aliphatic dicarboxylic acid to prepare semi-aromatic PBI is necessary for the current development of PBI applications. Moreover, another objective is to increase the mechanical strength by either chemically or physically approaches, for example, by crosslinking. In this work, a semi-aromatic polybenzimidazole (DPBI) was successfully synthesized via polycondensation of decanedioic acid (DCDA) and 3,3-diaminobenzidine (DAB) with phosphorus pentoxide/methanesulfonic acid (P_2_O_5_-MeSO_3_H, PPMA) as condensation agent and solvent. Subsequently, to improve the mechanical properties, the DPBI-*x*PVBC with crosslinked structure were prepared by using poly (vinylbenzyl chloride) (PVBC) as a macromolecular crosslinker during the membrane fabrication. The optimized DPBI-*x*PVBC membranes show the enhanced H_2_/O_2_ single cell performance at 160 °C without further humification, indicating its feasibility as HT-PEM for HT-PEMFCs.

## 2. Experimental

### 2.1. Materials

3,3′-Diaminobenzidine (DAB, 99%) was purchased from Sigma-Aldrich Corporation (Shanghai, China). Decanedioic acid (DCDA, 99%), phosphoric acid solution (85 wt.%) were obtained from Aladdin Chemistry Co. Ltd (Shanghai, China). Phosphorus pentoxide (P_2_O_5_, 98%), methanesulfonic acid (MeSO_3_H), poly (vinylbenzyl chloride) (PVBC, Mw = 20,000~50,000) and *m*-cresol (98%) were acquired from Macklin Biochemical Co. Ltd (Shanghai, China). All the materials were used as received.

### 2.2. Methods

#### 2.2.1. Synthesis of Semi-Aromatic Polybenzimidazole (DPBI)

**Scheme 1:**
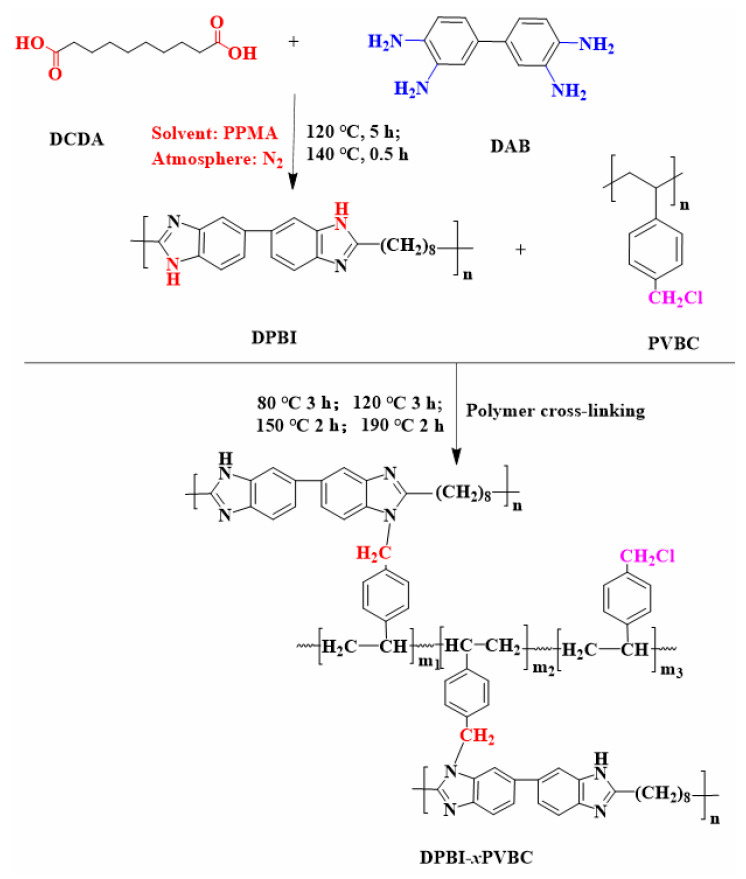
Preparation procedure of semi-aromatic DPBI based on dicarboxylic acid and cross-linked DPBI-*x*PVBC membranes.

DPBI was synthesized by solution polycondensation reaction of DCDA and DAB with PPMA as condensating agent and solvent according to the following procedure. In a 100 mL three-neck round-bottomed flask was filled with 3.75 g P_2_O_5_ and 37.5 g MeSO_3_H and equipped with a mechanical stirrer and a nitrogen inlet and outlet. The mixture was stirred at 80 °C for approximately 1 h, until a transparent solution was obtained. DCDA (1.0117 g, 5 mmol) was added and stirred at this temperature to dissolve it completely. Then, DAB (1.0716 g, 5 mmol) was added and stirred for 1 h, the reaction temperature was promoted to 120 °C and kept for 5 h. The reaction mixture was raised to 140 °C and continuously stirred for another 0.5 h. The final viscous solution was slowly poured into deionized water so as to make the polymer precipitated. The obtained fibrous polymers were crushed by a high-speed blender, washed with water and filtered. The polymer was stirred in NaHCO_3_ solution (10 wt.%) to neutralize the residual acid in the polymer, and then filtered and washed with water and acetone, respectively, till the pH of the filtrate is neutral. Finally, it was dried in an oven at 120 °C for 24 h. The synthesis process is shown in Scheme 1.

#### 2.2.2. Preparation of Crosslinked DPBI-xPVBC Membranes

As presented in Scheme 1, the DPBI-*x*PVBC membrane with crosslinked three-dimensional (3D) network was prepared by typical solution casting method and in situ crosslinking reaction. Both DPBI and PVBC powders were dissolved separately in *m*-cresol to form 5 wt% solutions under magnetic stirring at 140 °C and 80 °C, respectively. The two solutions were mixed in a given mass ratio and magnetically stirred for 1 h at room temperature. The produced mixture was directly poured onto a clean glass plate, glass rod was used to spread the solution evenly. Then the cross-linked membrane was obtained by a multi-step heating process (80 °C, 3 h; 120 °C, 3 h; 150 °C, 2 h; 190 °C, 2 h). The resultant membranes were named as DPBI-*x*PVBC, where *x* represents the weight percentage of PVBC in membrane. As a control membrane, the pristine DPBI membrane was also prepared by the same solution casting method. All the membranes were dried in an oven at 100 °C for 24 h to remove moisture before use.

### 2.3. Characterization and Measurements

#### 2.3.1. Structure Characterization and Morphology

^1^H nuclear magnetic resonance (^1^HNMR) analysis was carried out on a Bruker DRX-500 spectrometer (PerkinElmer, Inc., Waltham, MA, USA) using tetramethylsilane (TMS) as the internal standard, and deuterated dimethyl sulfoxide (DMSO-*d*_6_) as solvent, respectively. The Flourier transform infrared (FT-IR) spectroscopy of membrane was collected on an attenuated total reflection FT-IR spectrometer (ATR-FT-IR, Scientific Nicolet 6700t, Waltham, MA, USA) with a wavenumber range of 4000–650 cm^−1^. The molecular weight of the as-prepared DPBI polymer was calculated by measuring the viscosity of polymer solution using a Ubbelohde viscometer at 30 °C, and the concentration of polymer solution using *m*-cresol as the solvent was controlled at about 5 mg mL^−1^. The surface and cross-section morphologies of the membranes were measured using a scanning electron microscopy (UHR FE-SEM, SU8010, Hitachi Ltd., Tokyo, Japan).

#### 2.3.2. Gel Fraction Measurement

Gel fraction of the crosslinked DPBI-*x*PVBC membranes in the organic solvent were estimated through monitoring the weight loss by immersing the membrane in *m*-cresol at 80 °C for 12 h so as to obtain the crosslinking degree. The pristine DPBI membrane was also tested as a control. The gel fraction of DPBI-*x*PVBC membrane can be calculated by using the following equation:

(1) $$Gel\;fraction\;\left(\%\right)=W_{2}/W_{1}\times 100$$

where *W*_1_ and *W*_2_ are the weights of dry membranes before and after testing, respectively.

#### 2.3.3. PA Doping Content and Proton Conductivity

The PA doping content of all the membranes were obtained by immersing the membrane in 85 wt.% PA for a controlled time at room temperature. Before PA doping, the membrane samples were dried in an oven at 100 °C for 24 h, and their weight were recorded as *W_dry_*. After PA doping, the excess PA remained on the sample surface was wiped off with tissue paper, and its weight was denoted as *W_wet_*. To ensure the accuracy of the results, three samples were prepared for each membrane to get the average value. Finally, the PA doping content (ADC%) of membranes and volumetric swelling ratio is calculated in Equations (2) and (3) based on the weight and volume gains of membrane samples.

(2) $$ADC\;\left(\%\right)=\frac{W_{wet}-W_{dry}}{W_{dry}}\times 100\;$$

(3) $$Swelling\;ratio\;\left(\%\right)=\frac{V_{wet}-V_{dry}}{V_{dry}}\times 100\;$$

The proton conductivity was measured by a four-probe AC impedance method with an Autolab PGSTAT204 electrochemical workstation in the frequency range of 0.1 Hz to 105 Hz, and an oven was used to control the testing temperature (80 °C~180 °C). The proton conductivity (σ) was determined on the Equation (3):

(4) $$\sigma =\frac{L}{R\times A}$$

where *L*, *R* and *A* are the thickness, ohmic resistance and cross-sectional area of the membrane, respectively. The ohmic resistance was obtained from the Nyquist plots. In this work, all the proton conductivities were measured as proton transport in the direction of the membrane thickness.

#### 2.3.4. Mechanical Property

The mechanical property of the PA doped membrane was measured by employing a mechanical strength machine (CM640, Shenzhen, China). The stress-strain curves were analyzed at a constant elongation speed of 5 mm min^−1^ at room temperature in air atmosphere, and the size of the membrane samples was 40–50 mm in length and 4–6 mm in width. All results obtained came from the average value of at least three samples.

#### 2.3.5. Oxidative Stability

To reflect the durability of the membranes, the oxidative stability of the PA doped membrane was investigated by Fenton’s test (3 wt.% H_2_O_2_ solution with 4 ppm Fe^2+^) at 80 °C for 48 h. The oxidative stability (OS, %) was estimated as the remaining weight of all the membranes after testing by Equation (4). In order to guarantee the accuracy of the data, three samples of each membrane were taken for the experiment to obtain the average value.

(5) $$OS\;\left(\%\right)=W_{4}/W_{3}\times 100$$

where *W*_4_ and *W*_3_ are the weights of membrane samples before and after soaking, respectively.

#### 2.3.6. Fuel Cell Test

The membrane electrode assemblies (MEAs) were fabricated by a combination of gas diffusion electrodes (GDEs, Hensen Company, 1.0 mg cm^−2^ Pt/C) with Pt catalyst (0.4 mg cm^−2^ Pt loading) and the PA doped PEM without hot pressing. The active area and Pt loading of each electrode were 4 cm^2^ and 0.4 mg cm^−2^, respectively. Components of a single cell similar with sandwich structure include two metallic polar plates with single serpentine flow-fields, PET gaskets, MEA, which were stacked in sequence and fixed together by bolts. The fuel cell test was performed with a single cell testing system at 160 °C at a constant flow rate of 80 mL min^−1^ and 160 mL min^−1^ for H_2_ and O_2_ without humidification under ambient pressure, respectively. The polarization curve was recorded and controlled using a fuel cell testing system equipped with operation control software and an electronic load unit controller, which was purchased from Dalian Yuke Innovation Technology Co., Ltd., Dalian, China. For a short-term durability test, the single cell was loaded with a constant current density of 150 mA cm^−2^ at 140 °C for 50 h. Before this durability test, the cell was employed without further activation.

## 3. Results and Discussion

### 3.1. Structure Characterization of DPBI and DPBI-xPVBC

**Figure 1:**
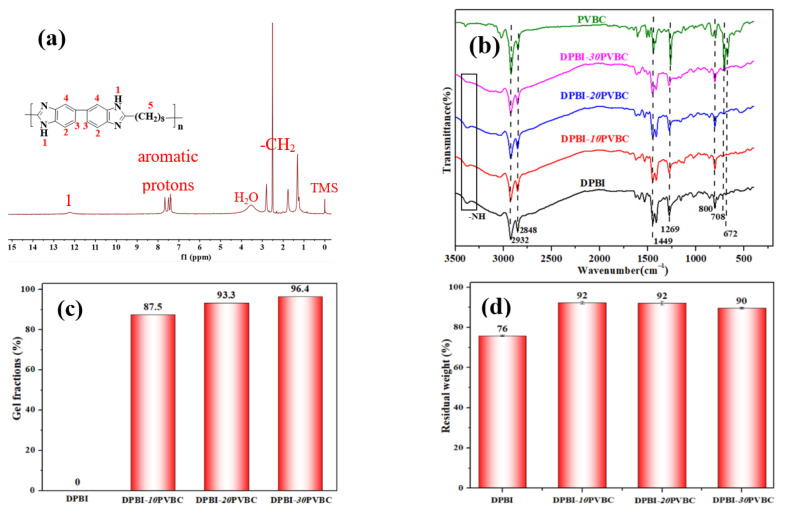
(**a**) 1H NMR spectra of DPBI in DMSO-*d*6, (**b**) IR spectroscopy, (**c**) gel fractions, (**d**) oxidative of DPBI and DPBI-*x*PVBC membranes.

In order to identify the viscosity-average molecular weight of DPBI polymer, the flow time of solvent and solution was measured in sequence with an Ubbelodhe viscometer. The viscosity-average molecular weight of DPBI polymer is 2.88, showing a high molecular weight. The chemical structure of DPBI were determined by ^1^H NMR spectra, as shown in Figure 1a. All characteristic proton signals of imidazole denoted as H_1_, biphenyl denoted as H_2_, H_3_, H_4_, and methylene denoted as H_5_ can be observed. The peak of a chemical shift between 12 and 13 ppm is attributed to the proton characteristic of the imidazole ring. The peaks with chemical shift of 7–8 ppm are ascribed to the protons of the benzene. The peaks in the 1–3 ppm are assigned to the methylene hydrogen on the semi-aromatic chain. The ^1^H NMR results confirm the successful synthesis of DPBI.

In addition, in terms of structural characterization, the FT-IR spectra of DPBI-*x*PVBC, DPBI and PVBC are compared in Figure 1b. The most interrelated absorption peaks are observed at 1269, 708 and 672 cm^−1^ for the chloromethyl group of PVBC, and the adsorption peak at 1269 cm^−1^ is also consistent with −C-N- of imidazole ring. The peaks at 1449 cm^−1^ and 800 cm^−1^are concerned in the −C=C- and −C=N- in-plane stretching vibration of PVBC and DPBI, and the peaks at 3400~3100 cm^−1^ is ascribed to the tensile vibration of N-H. In addition, there are two obvious peaks at 2932 cm^−1^, 2848 cm^−1^, matching with the stretching vibration peak of the C-H backbone. The characteristic absorption peaks described above distinctly confirms the successful in situ crosslinking reaction between DPBI and PVBC during fabricating the DPBI-*x*PVBC membranes.

The valid crosslinking reaction is confirmed via the solubility test in *m*-cresol for DPBI-*x*PVBC. The DPBI-*30*PVBC membranes have high gel content after treatment in *m*-cresol for 12 h at 80 °C, as depicted in Figure 1c. The gel contents of the DPBI-*10*PVBC and DPBI-*20*PVBC membranes are 93% and 88%, implying that the crosslinking reaction are successfully occurred between the chloromethyl groups of PVBC and the =N-H groups of benzimidazole and the highly crosslinked structure of DPBI-*x*PVBC are confirmed.

The oxidative stability is a pivotal factor affecting the performance of HT-PEMs. Due to the continuous provision of pure O_2_ at the cathode side, the PEM of HT-PEMFCs must have high oxidative stability. In addition, the radicals, including peroxyl (OOH) and hydroxyl (OH) can attack the polymer chains and result in the PEM degradation [30]. As depicted in Figure 1d, the pristine semi-aromatic DPBI membrane has the maximum weight loss of about 24 wt.% after Fenton’s test for 48 h at 80 °C, suggesting the low oxidative stability of DPBI. While the DPBI-*x*PVBC membranes exhibit only a slight weight loss after test, and their remain weights are 92%, 92%, 90% for the DPBI-*10*PVBC, DPBI-*20*PVBC and DPBI-*30*PVBC membranes, respectively, manifesting an admissible antioxidant durability of the crosslinked DPBI-*x*PVBC membranes. It is believed that the ·OH and HOO· radicals attack the hydrogen atom of N-H and C-H bonds of PBI backbone, and lead to the degradation of HT-PEMs [31,32,33]. Within a certain range, both the DPBI-*x*PVBC membranes with crosslinked structure show high oxidative stability, whose residue weights are higher than the pristine DPBI during the Fenton’s test, showing that the crosslinked 3D network is beneficial for improving the stability. Compared with DPBI-*10*PVBC and DPBI-*20*PVBC membranes, the DPBI-*30*PVBC shows slightly low residue weight after Fenton’s test. The higher content of crosslinker PVBC gives higher gel content but lower oxidative stability to DPBI-*30*PVBC compared with DPBI-*10*PVBC and DPBI-*20*PVBC. Presumably, with increasing the PVBC content, the benzyl chloride cannot react completely with the =N-H groups of imidazole units in DPBI, and the residue benzyl chloride groups are unstable and can produce benzyl radicals easily, accelerating the degradation of the membrane. Considering about the crosslinking degrees, PA uptake ability and oxidative stability, the content of the macromolecular crosslinker should be controlled below 30% for using as HT-PEMs. Additionally, the oxidation stability test of PA doped membranes was also tested in order to be closer to the conditions of reliable to fuel cells. According to the test phenomenon, PA doped DPBI membrane started to break into pieces after immersing in Fenton reagent for nearly 2 h, while PA doped DPBI-*10*PVBC started to break into pieces after immersing in Fenton reagent for nearly 4 h. In comparison with the pristine DPBI membrane, the notable improvement in antioxidant performance of the crosslinked DPBI-*x*PVBC membrane could be put down to the reduction of N-H content in the crosslinked membrane, which enhanced the resistance to free radical attack.

### 3.2. Performance Analysis of the Crosslinked DPBI-xPVBC Membranes

The proton conductivity of HT-PEM is a crucial peculiarity for HT-PEMFC application, even though the PEM exhibits high stability and good conformation, its low proton conductivity will place restrictions on its cell performance. For the PA doped HT-PEMs, since proton transport is fulfilled by means of the dissociation of PA molecules, high ADC is a prerequisite to achieve high proton conductivity. However, due to the “plasticizing effect” of the doped PA, the superabundant ADC will bring about the deterioration of mechanical properties of the membranes. The PA adsorbed speed of the common PBI based membrane is low and it need long time to achieve an equilibrium adsorption, resulting in the difficulty for controlling the ADC. Compared with *m*-PBI, the semi-aromatic DPBI herein has softer aliphatic unit in the polymer chain, which make it be able to adsorb PA very quickly. Combining the macromolecular-structure design and post-crosslinking strategy, the crosslinked DPBI-*x*PVBC membranes are developed to solve the trade-off between the proton conductivity and mechanical strength of HT-PEMs.

Firstly, ADC values of DPI membrane are 123%, 165% and 237% for the different immersing time of 0.5 min, 1 min and 2 min, in turn. It is thus clear that the DPBI membrane possesses the characteristics of the high PA adsorption capacity and fast adsorption rate, achieving the effect of instantaneous adsorption of PA. So, it is easy to control the PA adsorption capacity by adjusting the temperature and time. Initially, multiple DPBI membranes with ADC at or around 100% were selected to assess the proton conductivity and mechanical property, and the results were summarized in Table 1.

**Figure 2:**
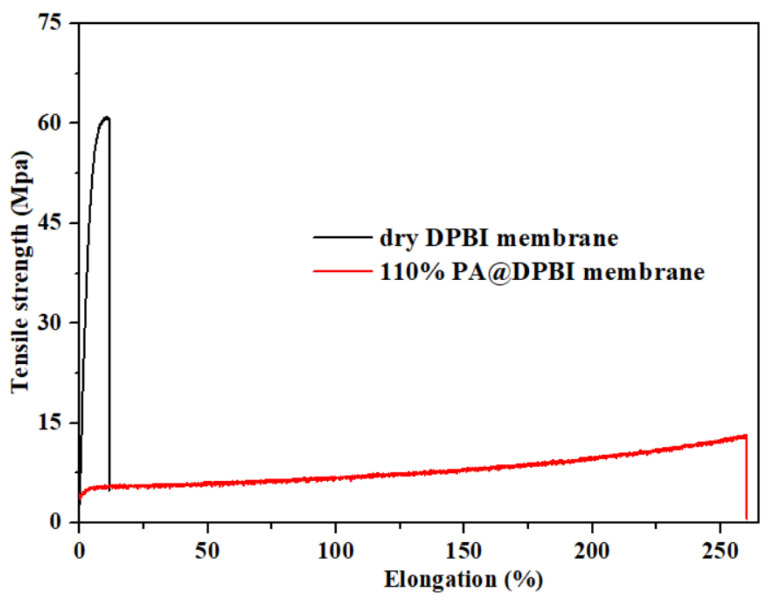
Stress-strain curves of the dry and PA doped DPBI with 110% ADC.

The stress strain curves of the dry and PA doped DPBI with 110% ADC are shown in Figure 2. The dry DPBI membrane shows a high tensile strength of 61.0 MPa and an elongation at break of 11.6%. After doping with PA, the 110%PA@DPBI membrane has tensile strength of only 13.2 MPa and high elongation at break of 260%. Meanwhile, the 110%PA@DPBI membrane exhibits a low proton conductivity of 19 mS cm^−1^ at 160 °C. Because of the strong plasticizing effect of PA for the DPBI membrane, it is impossible to promote the proton conductivity by increasing the PA doping level.

**Scheme 2:**
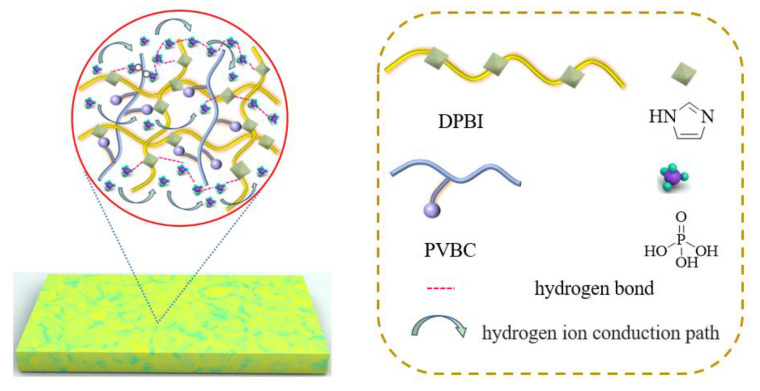
Schematic of proton transport of PA@ DPBI-*x*PVBC membrane.

**Figure 3:**
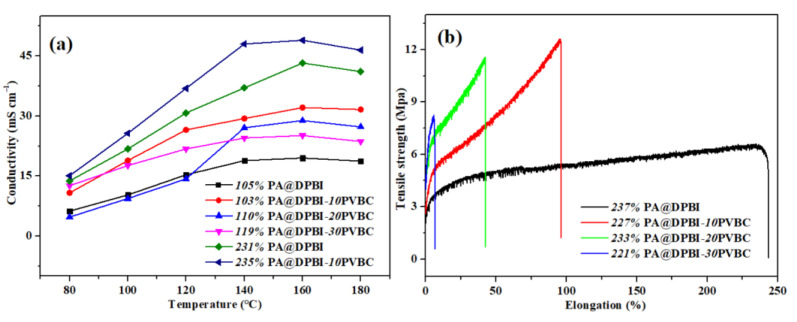
(**a**) Proton conductivities and (**b**) stress-strain curves of PA doped membranes.

The *y%*PA@DPBI-*x*PVBC membranes with different ADCs were obtained by adjusting the immersion time in PA (*y%* refers to the ADC value of the membrane), and their proton conductivities as a function of the temperature without humidification and their mechanical properties at ambient environment were also assessed, as revealed in Figure 3. As seen in Figure 3a, the in-plane proton conductivity of the entire PA doped DPBI *x*PVBC membranes increase with the increase of the temperature. Whereas a plateau emerges at 160 °C, resulting from the PA dehydration in the range of 160 °C to 180 °C [34,35]. For example, with increasing temperature from 80 °C to 160 °C, the proton conductivity of *103%* PA*@* DPBI-*10*PVBC varies from 11 mS cm^−1^ to 32 mS cm^−1^. Here, a schematic diagram is presented in Scheme 2 to explain the proton transport mechanism under the anhydrous conditions. It reveals that there are hydrogen bonds between PA molecules and imidazole rings, generating effective and continuous proton transport channels in accordance with Grotthuss mechanism. For the crosslinked membranes with the similar ADC, the DPBI-*10*PVBC membrane has the highest proton conductivity at the same temperature, due to the decrease content of DPBI with the increase of the PVBC content. In comparison with DPBI-*20*PVBC and DPBI-*30*PVBC membranes, the DPBI-*10*PVBC membrane has more imidazole groups, which can facilitate PA molecules forming more hydrogen-bonding networks for proton conduction. At the same time, considering that the PA doped HT-PEMs require higher ADC to obtain high proton conductivity at the anhydrous condition, the *235%*PA@DPBI-*10*PVBC membrane seems to be a good choice. As depicted in Figure 3a, the proton conductivity of the DPBI-*10*PVBC membrane increases from 32 mS cm^−1^ to 49 mS cm^−1^ when ADC increases from 103% to 235% at 160 °C.

The excellent mechanical performance of HT-PEMs is one of the key points for long-term stable operation of HT-PEMFCs. Xiao et al. reported that the polymer electrolyte membranes with a tensile strength greater than 1.5 MPa can be applied in fuel cell performance testing [36]. Therefore, we should adhere to the principle that the PA doped membrane occupy superior mechanical properties on the premise that the proton conductivity meets the requirements. Figure 3b presents the typical stress-strain curves of various PA doped crosslinked membranes, and the detail relevant values are also illustrated in Table 2. As shown in Figure 3b and Table 2, the *227%*PA@DPBI-*10*PVBC membrane exhibits the tensile strength and elongation at break of 12.6 MPa and 96.1%, respectively, which is much better than that of the DPBI membrane with the equivalent ADC. The enhanced mechanical properties of the crosslinked membrane are resulted from the low swelling ratio after the crosslinked reaction. Besides, it is noticed that the swelling ratio increased with the decrease of the PVBC content. It is worth noting that the high crosslinking degree would and make the membrane stiffer and even brittle [37]. The mechanical properties of the as-prepared crosslinked membrane confirm this expected tendency. After comprehensive consideration, the PA doped DPBI-*10*PVBC membrane was selected for subsequent fuel cell performance and durability test.

**Figure 4:**
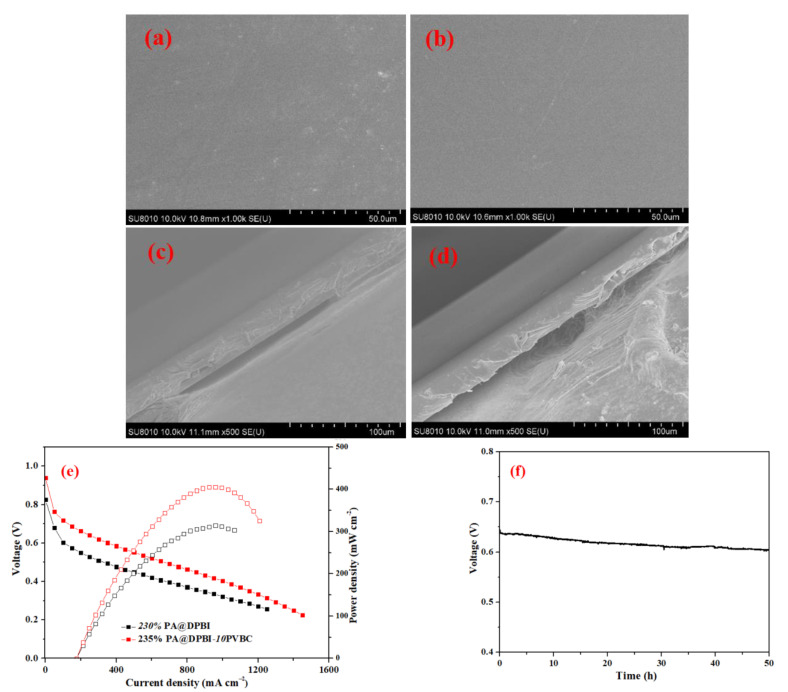
SEM micrographs of the surface (up) and cross-section (down) of the membranes: (**a**,**c**) DPBI, and (**b**,**d**) DPBI-*10*PVBC; (**e**) Polarization curves at 160 °C with flow rates of 80- and 160-mL min-1 for un-humidified H2 and O_2_ and (**f**) short time durability test at 150 mA cm^−2^ and 140 °C without humidification and backpressure of single cell with *235%*PA@DPBI-*10*PVBC.

Before testing the cell performance, the surface and cross section morphologies of DPBI and DPBI-*10*PVBC membranes were characterized by FE-SEM. In Figure 4a–d, both membranes show a completely uniform and compact structure with no pores, which is critical for separating fuel and oxidant during the cell operation and benefits fuel cell performance. The polarization curve of a single cell with the *235%*PA@DPBI-*10*PVBC membrane at 140 °C without humidification and backpressure is displayed in Figure 4e. First, the open circuit voltage is up to 0.94 V, suggesting that the PEM has remarkable gas barrier properties. Secondly, the highest power density (405 mW cm^−2^), which is much higher than that of the pristine DPBI membrane (314 mW cm^−2^). Additionally, it is comparable or even better HT-PEMFC performance than that of some recent reports for *m*-PBI and some other analogues containing PBI, as listed in Table 3. It is mainly credited to the excellent proton conductivity and better mechanical strength. Furthermore, a short-term dynamic test of the 235% PA@DPBI-*10*PVBC was also monitored to assess the reliability of the single HT-PEMFC, and the cell voltage was plotted as a function of time. As revealed in Figure 4f, the voltage drops from ~0.644 V to ~0.605 V during the test period of 50 h, and the voltage attenuation rate is 0.78 mV h^−1^. The improved performance of the single cell can be attributed to a result of the crucial contribution of its suitable proton conductivity and excellent mechanical property resulting from the crosslinking structure. The above results imply a potential application of the DPBI-*x*PVBC crosslinking membrane in HT-PEMFCs.

## 4. Conclusions

DPBI, a semi-aromatic PBI with enhanced comprehensive fuel cell performances was successfully synthesized. Furthermore, a series of crosslinked DPBI-*x*PVBC HT-PEMs can be then obtained with excellent antioxidation stability, high proton conductivity and improved mechanical strength using PVBC macromolecular crosslinker by a simple in situ crosslinking reaction. The crosslinked DPBI-*10*PVBC membrane with a gel fraction of 88% and an ADC of 235% displays the highest conductivity of approximately 49 mS cm^−1^ at 160 °C and the highest tensile strength of 12.6 MPa at room temperature. Meantime, the maximum power density of the HT-PEMFC with PA doped DPBI-*10*PVBC membrane can reach 405 mW cm^−2^, with an average voltage attenuation of 0.78 mV h^−1^ during the short-term stability test of 50 h. In summary, the crosslinked DPBI-*x*PVBC PEM shows a promising application in HT-PEMFCs.

## Figures and Tables

**Table 1:** Proton conductivities and mechanical properties of PA undoped DPBI and DPBI membranes with ADC around 100%.

Membrane	ADC /%	σ/mS cm^−1^ (160 °C)	Tensile Strength/MPa	Elongation (%)
Dry DPBI	0	-	58.7 ± 2.2	9.2 ± 2.4
110%PA@DPBI	110	19.4 ± 0.1	14.2 ± 1.1	261.0 ± 1.0

**Table 2:** PA uptake, and mechanical properties of PA doped DPBI-*x*PVBC membranes.

Membrane	ADC (%)	S_volume_ (%)	Tensile Strength (MPa)	Elongation (%)
PA@DPBI	237 ± 3	111 ± 12	7.00 ± 0.4	269 ± 28.3
PA@DPBI-*10*PVBC	227 ± 3	83 ± 2	15.0 ± 2.4	118 ± 22.1
PA@DPBI-*20*PVBC	231 ± 3	81 ± 1	11.2 ± 1.0	26.3 ± 16.2
PA@DPBI-*30*PVBC	221 ± 3	76 ± 3	8.00 ± 0.5	6.30 ± 0.9

**Table 3:** Summary comparison of HT-PEMFCs with PA doped PBI based membrane.

Membrane	ADLs	ADC (%)	Conductivity (mS cm^−1^)	OCV (V)	Power Density (mW cm^−2^)	Fuel Gas	Temperature (°C)	Ref.
DPBI-*10*PVBC		235	49 (160 °C)	0.94	405	H_2_/O_2_	160	This work
SC-B-OPBI-10		223	44 (180 °C)	0.88~0.94	404	H_2_/air	160	[38]
CrL-4.6%F_6_ PBI	13.5		115 (160 °C)	1.00	360	H_2_/air	160	[39]
g-PBI-30	24.7		178 (160 °C)	0.87	383	H_2_/O_2_	160	[40]
HB-PBI		331	168 (150 °C)	0.82	346	H_2_/O_2_	150	[41]
F_6_-oxyPBI-2SO_3_H			49 (120 °C & 50%RH)	0.90	360	H_2_/air	150	[42]
m-PBI		375	-	1.00	352	H_2_/air	160	[43]
Be_3_Br-7.5%	11.6		95 (160 °C)	0.95	340	H_2_/O_2_	160	[44]
PBI/GO-1	10.0		130 (165 °C)	0.95	380	H_2_/air	165	[45]
c-6-s-TiO_2_-PBI-OO		392	98 (160 °C)	1.00	356	H_2_/air	160	[46]
OPBI/PIM-1(10%)	18.7		313 (200 °C)	0.94	438	H_2_/O_2_	160	[47]

## Data Availability

Not applicable.
